# ***Akkermansia muciniphila*** **modulates intestinal mucus composition to counteract high-fat diet-induced obesity in mice**

**DOI:** 10.1080/19490976.2025.2612580

**Published:** 2026-01-09

**Authors:** Paola Paone, Camille Petitfils, Anthony Puel, Dimitris Latousakis, Willem M. de Vos, Nathalie M. Delzenne, Nathalie Juge, Matthias Van Hul, Patrice D. Cani

**Affiliations:** a Metabolism and Nutrition Research Group, Louvain Drug Research Institute, UCLouvain, Université catholique de Louvain, Brussels, Belgium; b Walloon Excellence in Life Sciences and BIOtechnology (WELBIO), WELBIO Department, WEL Research Institute, Wavre, Belgium; c Quadram Institute Bioscience, Food, Microbiome and Health Institute Strategic Programme, Norwich Research Park, Norwich, UK; d Laboratory of Microbiology, Wageningen University, Wageningen, The Netherlands; e Human Microbiome Research Program, Faculty of Medicine, University of Helsinki, Helsinki, Finland; f Institute of Experimental and Clinical Research (IREC), UCLouvain, Université catholique de Louvain, Brussels, Belgium; g Section of Biomolecular Medicine, Division of Systems Medicine, Department of Metabolism, Digestion and Reproduction, Imperial College London, London, United Kingdom

**Keywords:** Mucus, gut microbiota, *Akkermansia muciniphila*, diabetes, obesity, glycan profiles

## Abstract

**Objective:**

This study investigates whether live *Akkermansia muciniphila* Muc^T^ supplementation can counteract obesity and metabolic dysfunctions induced by a high-fat diet (HFD) by modulating intestinal mucus production, secretion and composition.

**Design:**

C57BL/6J mice were fed an HFD with or without live *A. muciniphila* Muc^T^ (2 × 10^8^ CFU per day) supplementation or a control diet for 6 weeks. Body weight, fat mass gain and metabolic markers were measured. Intestinal mucus characteristics were assessed via gene expression analysis of mucins and analysed mucin glycosylation by tandem mass spectrometry (MS/MS).

**Results:**

Mice receiving live *A. muciniphila* Muc^T^ exhibited reduced body weight gain and fat mass accumulation compared to HFD controls, without changes in muscle mass. *A. muciniphila* improved gut barrier integrity by increasing antimicrobial peptide expression in the jejunum and in the colon of HFD-fed mice. Furthermore, live *A. muciniphila* Muc^T^ influenced markers of goblet cell differentiation and restored the expression of mucin markers altered by HFD. Specifically, live *A. muciniphila* Muc^T^ counteracted HFD-induced mucin 3 (Muc3) expression depletion in the colon. Although the overall mucus thickness was not affected by live *A. muciniphila* Muc^T^, the bacteria significantly modulated mucin glycans composition. Live *A. muciniphila* Muc^T^ did not change the gut microbiota composition.

**Conclusion:**

These findings highlight the protective effects of live *A. muciniphila* Muc^T^ against diet-induced metabolic dysfunctions by modulating adiposity, mucus layer composition, and glycan profiles. This reinforces its potential as a therapeutic strategy for metabolic disorders associated with gut microbiota alterations.

## Introduction

Obesity and type 2 diabetes are complex metabolic disorders associated with gut barrier dysfunction.^1^^,^^2^ The intestinal mucus layer, which serves as a critical protective barrier between the gut microbiota and host epithelium, plays a crucial role in maintaining gut homeostasis.^3^ Disruptions to this barrier have been implicated in metabolic dysregulation and disease progression. Previous research from our group has shown that dietary interventions, including prebiotics such as oligofructose (FOS) or 2'-fucosyllactose (2'FL), can modulate mucus properties and protect against obesity and diabetes in murine models.^4^^,^^5^ These interventions have also been associated with significant shifts in cecal and fecal microbiota composition,^4^^,^^5^ notably increasing the relative and absolute abundance of *Akkermansia muciniphila*, a bacterium widely recognized for its metabolic benefits.

*A. muciniphila* is a prominent member of the gut microbiota that resides within the mucus layer, where it plays an essential role in host-microbiota interactions.^6-11^ As a mucin-degrading specialist, it colonizes and thrives within the mucus layer, where its activity paradoxically strengthens gut barrier integrity by stimulating mucin production and enhancing host defense mechanisms.^8^^,^^12^ The abundance of *A. muciniphila* has been found to be lower in the feces of obese and diet-induced obese mice, as well as in obese patients, compared to lean mice or healthy subjects.^8^^,^^11-13^

In rodent studies, supplementation with *A. muciniphila* has been shown to increase goblet cell numbers and mucus production, counteracting the adverse effects of a high-fat diet (HFD) on gut barrier integrity.^8,13‐15^ This was associated with improved metabolic outcomes including enhanced insulin sensitivity, reduced inflammation, and decreased fat mass, suggesting a potential causal relationship between *A. muciniphila* presence and metabolic health.

A few human trials have assessed the effects of *A. muciniphila* supplementation on metabolic health. The first human intervention, that spanned a 3-month period, demonstrated that both live and pasteurized forms of the bacterium are safe and well-tolerated in overweight and obese insulin-resistant individuals.^9-11^ *A. muciniphila* administration improved insulin sensitivity, reduced plasma cholesterol levels, slightly decreased body weight, fat mass and hip circumference and reduced blood markers of liver dysfunction and inflammation.^9‐11^ Overall gut microbiota composition was not affected and the extent to which these effects were driven by mucus modulation is unknown. A second human trial confirmed that *A. muciniphila* supplementation provides metabolic benefits though this study also highlighted that the colonization efficiency and clinical benefits of *A. muciniphila* may depend on baseline *A. muciniphila* abundance, suggesting that individual responses to supplementation may vary.^16^

Despite its recognized role in gut barrier function, none of the studies investigating *A. muciniphila* in the context of obesity and diabetes have directly examined its influence on markers of mucus production, glycosylation, and secretion. Given the critical role of the intestinal mucus layer in gut barrier integrity and metabolic regulation, understanding these interactions is essential. To address this gap, the present study investigates the impact of live *A. muciniphila* Muc^T^ supplementation on intestinal mucus production, secretion, and composition in different gut segments of HFD-fed mice. By providing a detailed characterization of the mucus composition and glycan profiles, this study aims to further elucidate the mechanisms through which live *A. muciniphila* Muc^T^ exerts its beneficial effects on host metabolism and gut homeostasis.

## Results

### Live *A. muciniphila* Muc^T^ counteracts metabolic alterations induced by HFD

**Figure 1.**
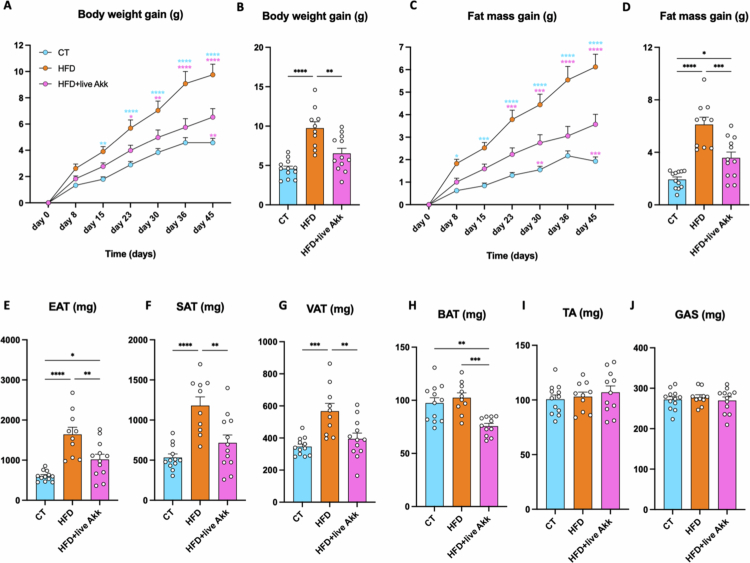
Administration of live *A. muciniphila* Muc^T^ counteracts diet-induced obesity. (A) Body weight gain evolution and (C) fat mass gain evolution. (B) Final body weight gain and (D) fat mass gain. (E–H) Adipose tissue weights of epididymal (EAT), subcutaneous (SAT), visceral (VAT) and brown (BAT) adipose tissue. (I–J) Muscle tissue weights of tibialis anterior (TA) and gastrocnemius (GAS). Data are means ± s.e.m (*n* = 10–12/group). One-way ANOVA followed by Tukey post hoc test was applied to figure B, D, E–H. Two-way ANOVA followed by Tukey post hoc test was applied to figure A, C. Data with different subscript letters are significantly different (*P* < 0.05). The presence of outliers was assessed using the Rout test. *P < 0.05; **P < 0.01; ***P < 0.001; ****P < 0.0001.

To investigate the effect of live *A. muciniphila* Muc^T^ on the adverse effects of diet-induced obesity, mice (*n* = 36) were split into 3 groups (*n* = 12) fed a control diet (CT), a HFD or HFD supplemented with live *A. muciniphila* Muc^T^ (HFD + live Akk). HFD feeding resulted in a significant increase in body weight compared to the CT group, while administration of live *A. muciniphila* Muc^T^ significantly reduced body weight gain. Similarly, live *A. muciniphila* markedly reduced HFD-diet induced fat mass gain and adipose tissue weights across multiple depots including epididymal (EAT), subcutaneous (SAT), visceral (VAT) and brown (BAT) adipose tissues. Muscle mass remained unchanged by the dietary interventions, indicating a selective effect on adiposity, as determined by the mass of tibialis anterior (TA) and gastrocnemius (GAS) (Figure 1).

### Live *A. muciniphila* Muc^T^ modulates the expression of barrier-related genes (Pla2g2a, Intectin) in the jejunum

**Figure 2.**
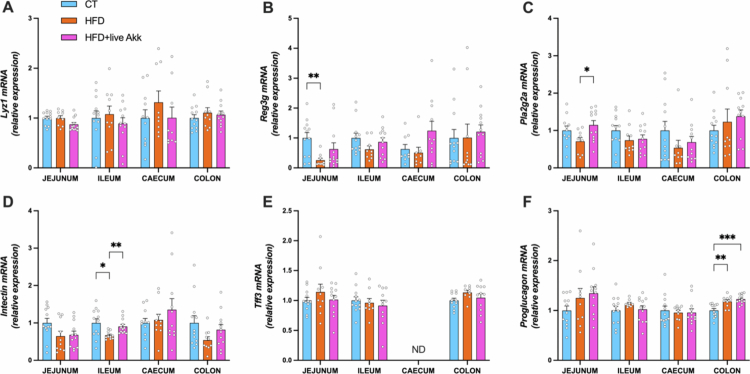
Live *A. muciniphila* Muc^T^ increases intestinal cells proliferation and markers involved in gut function. Relative mRNA expression of (A) Lysozyme C (*Lyz1*), (B) Regenerating islet-derived 3-gamma (*Reg3g*), (C) Phospholipase A2 group II (*Pla2g2a*), (D) Intectin, (E) Trefoil factor 3 (*Tff3*), and (F) Proglucagon across gut segments (jejunum, ileum, cecum, colon). Data are shown as mean ± s.e.m. (*n* = 8–12/group). Statistical analysis was performed using a mixed-effects model (REML) followed by Tukey’s multiple comparisons test. The presence of outliers was assessed using the Rout test. *P < 0.05; **P < 0.01; ***P < 0.001. ND = Not Detectable.

We analyzed the mRNA expression of antimicrobial peptides (lysozyme 1 (*Lyz1*), regenerating islet-derived protein 3-gamma (*Reg3g*), phospholipase A2 group IIa (*Pla2g2a*)), and markers involved in gut function (intectin, trefoil factor 3 (Tff3) and *proglucagon*) in the jejunum, ileum, cecum and colon. HFD feeding significantly reduced mRNA expression of *Reg3g* and *intectin*, a protein essential for epithelial cell turnover, in the jejunum and ileum, respectively. Live *A. muciniphila* Muc^T^ did not affect *Reg3g* expression (Figure 2B–D), but significantly increased *intectin* expression in the ileum and the antimicrobial peptides *Pla2g2a* in the jejunum HFD-fed mice (Figure 2C). Finally, HFD feeding and live *A. muciniphila* increased *proglucagon* expression in the colon, whereas none of the other markers were significantly affected in the different intestinal segments (Figure 2).

### Live *A. muciniphila* Muc^T^ modulates markers of goblet cell differentiation, mucus production and secretion in HFD-fed mice

**Figure 3.**
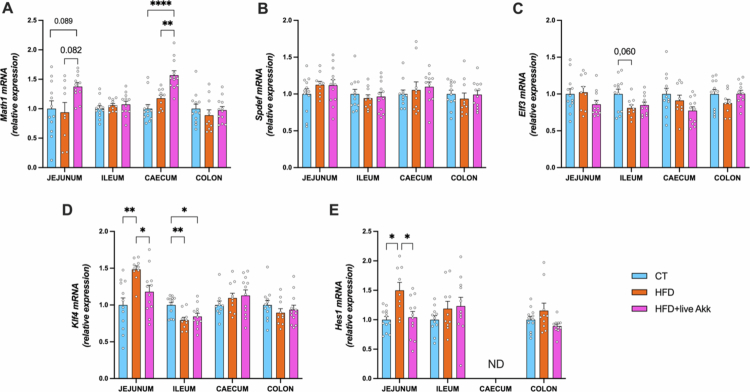
Live *A. muciniphila* Muc^T^ affects goblet cells differentiation. (A) atonal bHLH transcription factor 1 (*Math1*), (B) SAM pointed domain containing ETS transcription factor (*Spdef*), (C) E74 like ETS transcription factor 3 (*Elf3*), (D) Kruppel like factor 4 (*Klf4*), (E) Hes family bHLH transcription factor 1 (*Hes1*) mRNA relative expression of transcriptional factors involved in the goblet cells differentiation, in the jejunum, ileum, cecum and colon. Data are means ± s.e.m (*n* = 9-12/group). Statistical analysis was performed using a mixed-effects model (REML) followed by Tukey’s multiple comparisons test. The presence of outliers was assessed using the Rout test. *P < 0.05; **P < 0.01; ***P < 0.001; ****P < 0.0001. ND = Not Detectable.

Given its role as a mucus specialist, we investigated whether live *A. muciniphila* Muc^T^ could affect various stages of mucus production by measuring several mRNA markers linked to goblet cells, which produce and secrete the intestinal mucus layer. Additionally, we examined whether the effects of live *A. muciniphila* Muc^T^ supplementation on metabolism were linked to changes in markers of goblet cell differentiation. We found that live *A. muciniphila* Muc^T^ supplementation under HFD significantly influenced the expression of genes involved in stem cell differentiation into secretory cells,^17^ with increased expression of atonal bHLH transcription factor 1 (*Math1*) in the cecum and a trend towards significance in the jejunum (*P* = 0.08) (Figure 3A). The mRNA expression of transcription factor SAM pointed domain containing ETS transcription factor (*Spdef*) was unaffected by the different treatments (Figure 3B). In contrast, E74-like ETS transcription factor 3 (*Elf3*) expression tended to be reduced in the ileum of HFD-fed mice (*p* = 0.06) (Figure 3C). Importantly, live *A. muciniphila* Muc^T^ partially restored HFD-induced dysregulation of kruppel like factor 4 (*Klf4*) expression in the jejunum and ileum, as well as *Hes1* in the jejunum (Figure 3D and E), indicating a potential role in restoring goblet cell homeostasis.

**Figure 4.**
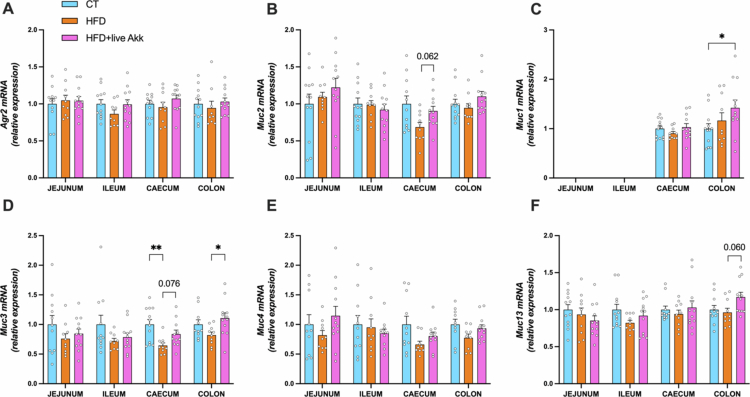
Live *A. muciniphila* Muc^T^ affects markers of mucus production. (A) Anterior gradient 2 (*Agr2*), (B) mucin 2 (*Muc2*), (C–F) mucin 1/3/4/13 (*Muc1*, *Muc3*, *Muc4, Muc13*) mRNA relative expression of markers involved in mucin production in the jejunum, ileum, cecum and colon. Data are means ± s.e.m (*n* = 6-12/group). Statistical analysis was performed using a mixed-effects model (REML) followed by Tukey’s multiple comparisons test. The presence of outliers was assessed using the Rout test. *P < 0.05; **P < 0.01; ***P < 0.001; ****P < 0.0001. ND = Not Detectable.

To determine the impact of live *A. muciniphila* Muc^T^ on intestinal mucins in HFD-fed mice, we assessed the expression of secreted and membrane-associated mucin encoding genes as well as anterior gradient 2 (*Agr2*), a key factor in the post-transcriptional synthesis and secretion of Muc2 in the colon.^18^ We found that *Agr2* and *Muc2* expression remained unchanged by the different treatments (Figure 4A,B) and that *Muc1* expression was increased by live *A. muciniphila* Muc^T^ supplementation in the colon (Figure 4C). Transmembrane mucins, which contribute to epithelial protection and intracellular signaling,^19^ were also affected by dietary interventions. HFD significantly decreased *Muc3* expression in the cecum and this tended to be corrected by live *A. muciniphila* Muc^T^ (*p* = 0.07). Likewise, live *A. muciniphila* Muc^T^ supplementation significantly increased *Muc3* expression in the colon compared to HFD-fed mice (Figure 4D) and showed a trend towards increased *Muc13* expression in the colon (*P* = 0.06) (Figure 4F). *Muc4* was not affected by the different treatments.

### Live *A. muciniphila* Muc^T^ influences markers of mucin secretion and stabilization in HFD-fed mice

**Figure 5.**
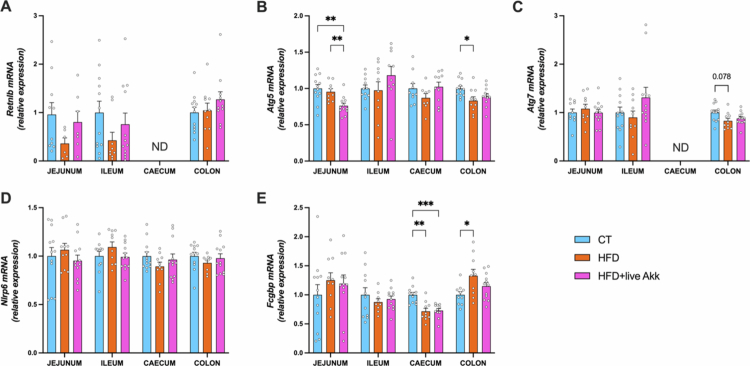
Live *A. muciniphila* Muc^T^ affects markers of mucus secretion. (A) Resistin-like beta (*Retnlb*), (B) Autophagy protein 5 (*Atg5*), (C) Autophagy protein 7 (*Atg7*), (D) NOD‐like receptor family pyrin domain containing 6 (*Nlrp6*), (E) Fc gamma binding protein (*Fcgbp*) mRNA relative expression of markers involved in the secretion of the mucus layer in the jejunum, ileum, cecum and colon. Data are means ± s.e.m (*n* = 6–12/group). Statistical analysis was performed using a mixed-effects model (REML) followed by Tukey’s multiple comparisons test. The presence of outliers was assessed using the Rout test. *P < 0.05; **P < 0.01; ***P < 0.001. ND = Not Detectable.

To investigate the effect of HFD on mucin secretion and assess live *A. muciniphila* Muc^T^ effects on stabilization, we analyzed several markers involved in mucus secretion and stabilization. HFD reduced, but not significantly, the expression of bactericidal protein resistin-like beta (*Retnlb*) in the upper intestines (jejunum and ileum) (Figure 5A). Additionally, HFD significantly reduced the expression of a key autophagy-related gene *Atg5* in the colon, while no differences were observed for *Atg7.* Of note the *Atg5* expression was decreased most after the live *A. muciniphila* Muc^T^ administration in the jejunum. *Fcgbp* expression decreased in the cecum and increased in the colon of HFD-fed mice (Figure 5B–E), suggesting region-specific alterations in mucus secretion and stabilization. It is worth noting that histological analysis of alcian blue-stained in colonic sections revealed that HFD feeding led to a significant increase in mucus layer thickness compared to control mice (Supplementary Figure 1) but no changes in the proportion of the blue area (representing the mucins) over the total mucosal area. Supplementation with live *A. muciniphila* Muc^T^ did not significantly alter these parameters. Although this finding appears counterintuitive given the improved barrier-related markers observed at the transcriptional level, it likely reflects compositional rather than volumetric changes in the mucus layer. Therefore, analyzing the mucin glycan profiles might be more relevant in this context.

### Live *A. muciniphila* Muc^T^ modifies mucin glycan profiles in HFD-fed mice

**Figure 6.**
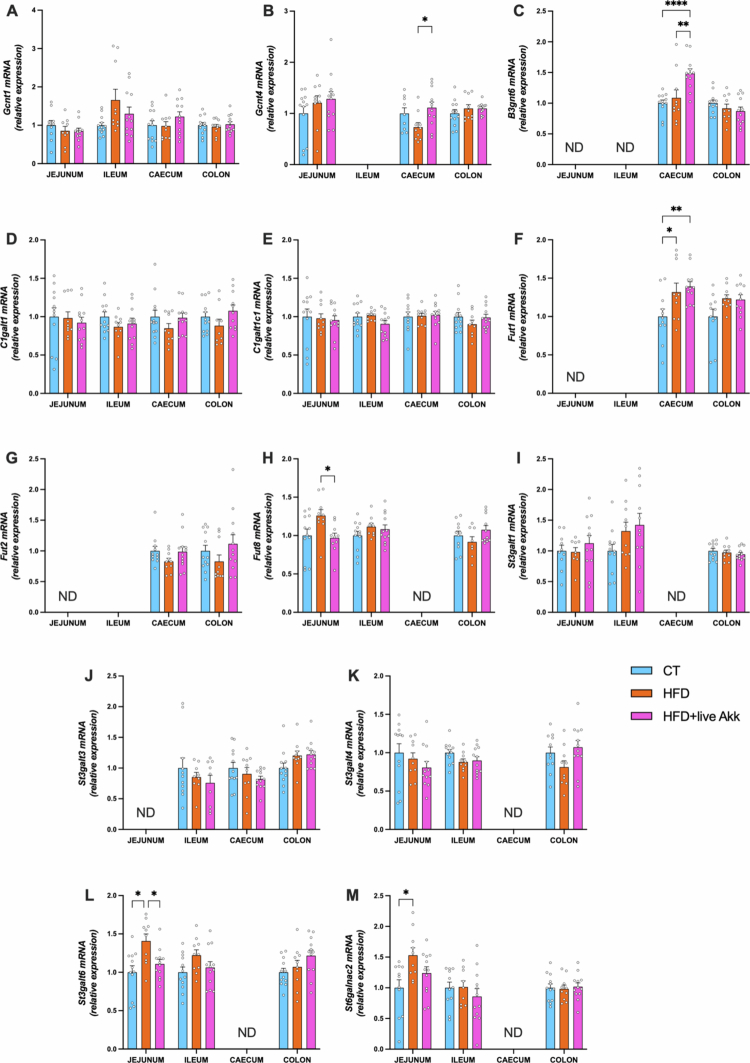
Live *A. muciniphila* Muc^T^ affects the expression of glycosyltransferases involved in mucin glycosylation. (A) glucosaminyl (*N*-acetyl) transferase 1 (*Gcnt1*), (B) glucosaminyl (*N*-acetyl) transferase 4 (*Gcnt4*), (C) UDP-GlcNAc:bGal b-1,3-*N*-acetylglucosaminyltransferase 6 (*B3gnt6*), (D) core 1 synthase, glycoprotein-*N*-acetylgalactosamine 3-b-galactosyltransferase 1 (*C1galt1*), (E) C1GALT1 specific chaperone 1 (*C1galt1c1*), (F–H) fucosyltransferase 1/2/8 (*Fut1*, *Fut2*, *Fut8*), (I-M) ST3 b-galactoside a-2,3-sialyltransferase 1/3/4/6 (*St3gal1*, *St3gal3*, *St3gal4*, *St3gal6*), ST6 *N*-acetylgalactosaminide a-2,6-sialyltransferase 2 (*St6galnac2*) mRNA relative expression of glycosyltransferases in the jejunum, ileum, cecum and colon. Data are means ± s.e.m (*n* = 8-12/group). Statistical analysis was performed using a mixed-effects model (REML) followed by Tukey’s multiple comparisons test. The presence of outliers was assessed using the Rout test. *P < 0.05; **P < 0.01; ***P < 0.001; ****P < 0.0001. ND = Not Detectable.

**Figure 7.**
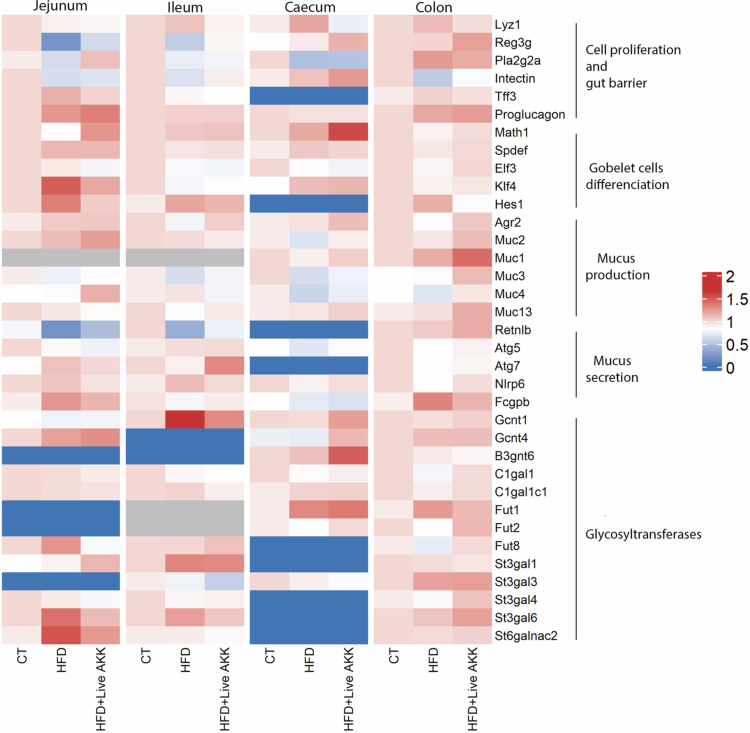
Heatmap of average gene expression levels across intestinal segments. The heatmap shows the group mean of relative gene expression levels from Figures 2–6, covering all intestinal segments (jejunum, ileum, cecum, and colon). Each cell represents the average relative expression of a given gene in a specific intestinal segment. Blue indicates genes that were not detected (ND) by qPCR, while gray corresponds to untested genes.

To determine whether HFD and *A. muciniphila* supplementation affected mucin glycosylation, we analyzed the expression of glycosyltransferases, enzymes responsible for attaching specific glycans to the protein backbone of mucins.^20^^,^^21^ Each glycosyltransferase catalyzes the addition of a particular glycan at a defined position, thereby shaping the structure and function of the mucus layer. By measuring the expression of glycosyltransferases involved in elongation and branching processes, we found no differences in expression of *N*-acetylglucosaminyltransferases *Gcnt1,* in the Galactose-transferase *C1galt1* (Figure 6D) and its specific chaperone *C1galt1c1* (Figure 6E), nor in the sialyltransferases *St3gal1* (Figure 6I)*, St3gal3* (Figure 6J) and *St3gal4* (Figure 6K)*.* However, we found a significant increase of the mRNA expression of sialyltransferases *St3gal6* and *St6galnac2* in the jejunum of HFD-fed mice (Figure 6L and M). These effects were abolished by live *A. muciniphila* Muc^T^ supplementation. In addition, live *A. muciniphila* Muc^T^ HFD**-**treated mice exhibited significantly elevated expression of *Gcnt4, B3gnt6* and the fucosyltransferase *Fut1* in the cecum (Figure 6B, C and F), coding for enzymes associated with glycan maturation and fucosylation. We observed that HFD tended to increase *Fut8* expression in the jejunum (*P* = 0.057) while live *A. muciniphila* Muc^T^ supplementation led to significant decreased *Fut8* expression compared to HFD-fed mice (Figure 6H). For the sake of clarity we summarized all the PCR data as an heatmap grouping all the mean relative gene expression levels from Figures 2–6 and covering all intestinal segments (jejunum, ileum, cecum, and colon) (Figure 7).

**Figure 8.**
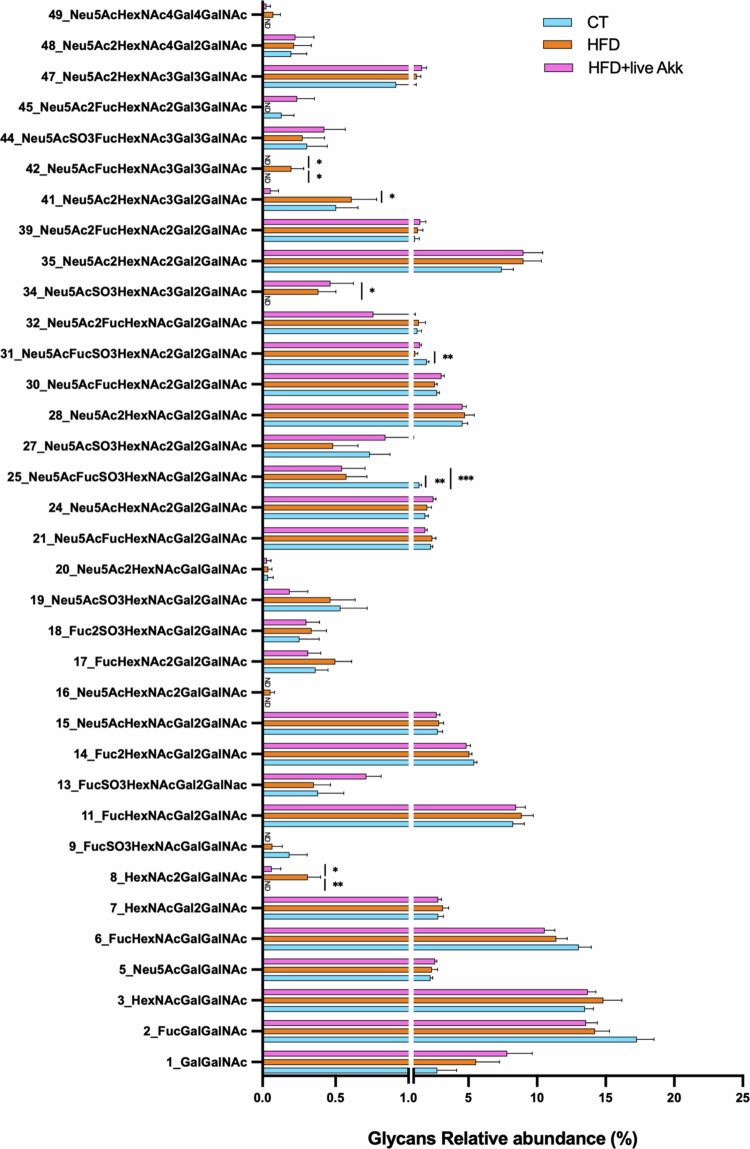
Live *A. muciniphila* Muc^T^ affects mucin glycan profile. Glycan relative abundance in percentage, sialylated glycans, fucosylated glycans and sulfated glycans. Data are means ± s.e.m (*n* = 9–10/group). One-way ANOVA followed by Tukey post hoc test or Kruskal-Wallis followed by Dunn’s test were applied based on data distribution. The presence of outliers was assessed using the Rout test. *P < 0.05. ND = Not Detectable.

Next, we performed MS/MS-based glycan analysis (Figure 8). The abundance of specific glycans was significantly altered in HFD-fed mice compared to CT mice. For example, Neu5AcFucHexNAc3Gal3GalNAc was exclusively detected in HFD-fed mice but absent in CT and live *A. muciniphila* Muc^T^*-*treated mice. Similarly, HexNAc2GalGalNAc was not detected in CT mice and was significantly decreased 5 fold in live *A. muciniphila* Muc^T^-treated mice (Figure 8). Conversely, Neu5Ac2FucHexNAc2Gal3GalNAc was undetectable in HFD-fed mice and Neu5AcSO3HexNAc3Gal2GalNAc was only detectable in the two HFD-fed groups of mice.

In addition, Neu5AcFucSO3HexNAcGal2GalNAc was significantly decreased in both HFD-fed groups compared to CT mice, while Neu5AcFucSO3HexNAc2Gal2GalNAc was significantly decreased in HFD-fed mice but was 30% higher in live *A. muciniphila* Muc^T^ treated mice. Among the different glycans, Neu5Ac2HexNAc3Gal2GalNAc was the only one significantly decreased by live *A. muciniphila* Muc^T^ without being affected by either CT or HFD treatment.

**Figure 9.**
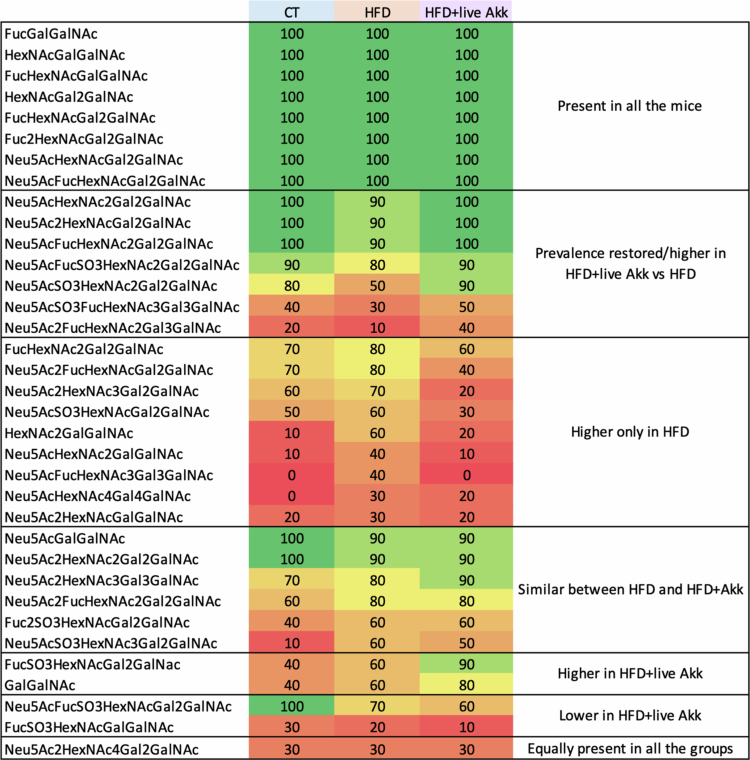
High-fat diet and live *A. muciniphila* Muc^T^ affects mucin glycan prevalence. Glycan prevalence calculated by dividing the number of mice for which the glycan was present for the total number of mice in the group. Data are means ± s.e.m (*n* = 10/group). Data were analyzed using Kruskal-Wallis followed by Dunn’s test. The presence of outliers was assessed using the Rout test. *P < 0.05. ND = Not Detectable.

Figure 9 depicts glycan prevalence calculated by dividing the number of mice for which the glycan was present for the total number of per group of mice, with 8 glycans being present in all three groups, irrespective of intervention (Figure 9). Another seven glycans showed decreased prevalence in HFD-fed mice and 9 showed increased prevalence in HFD-fed mice but the prevalence of these 16 glycans was restored by live *A. muciniphila* Muc^T^ (Figure 9). Finally, few other glycans were either similar between HFD and live *A. muciniphila* Muc^T^ treated mice or only affected by live *A. muciniphila* Muc^T^ (Figure 9).

### Effects of diet and live *A. muciniphila* Muc^T^ supplementation on gut microbiota diversity

**Figure 10.**
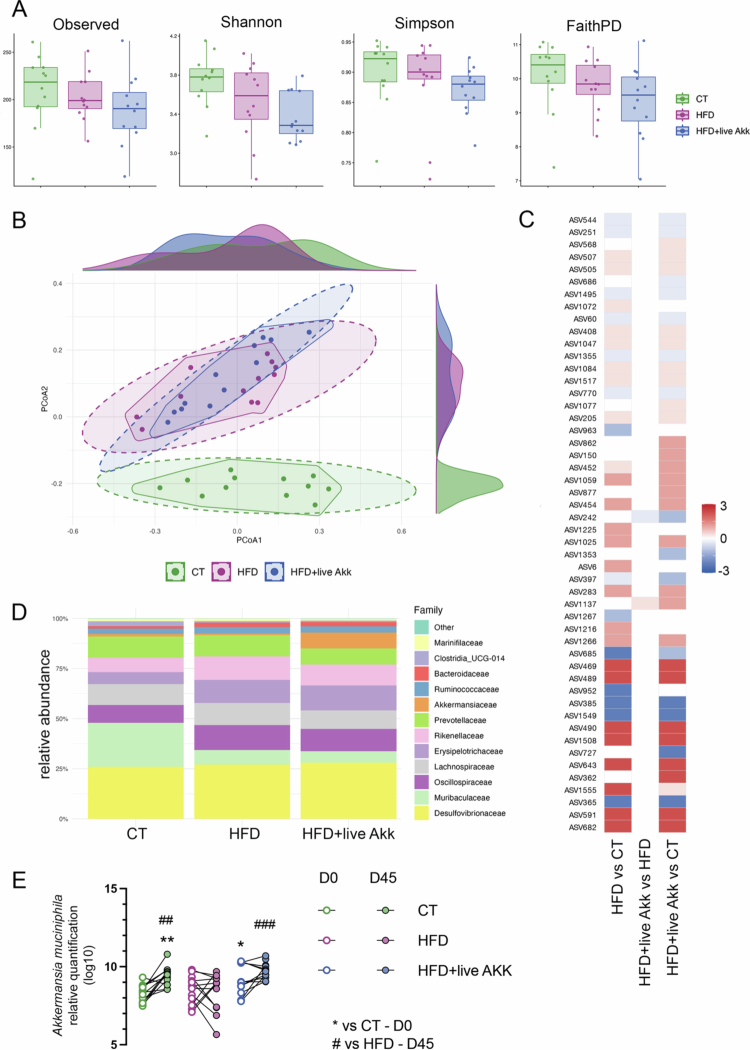
Effects of diet and live *A. muciniphila* Muc^T^ supplementation on gut microbiota diversity and composition. (A) Alpha diversity indices (Observed ASVs, Shannon, Simpson, and Faith’s phylogenetic diversity) showing no significant differences between groups. (B) Principal coordinates analysis (PCoA) with marginal densities based on Bray-Curtis distance illustrating distinct clustering by diet. PERMANOVA confirmed significant compositional differences between CT and HFD groups, and between CT and HFD + live Akk, while no separation was observed between HFD and HFD + live Akk. (C) Differential abundance analysis highlighting ASVs significantly associated with each condition. Scaled log₂ fold changes (heatmap) indicate taxa enriched or depleted in each pairwise comparison (ASV242 = *Akkermansia*, ASV1137 = unidentified *Lachnospiraceae*). (D) Family-level community composition showing major bacterial families across groups. (E) Relative quantification of *Akkermansia muciniphila* by qPCR at baseline (D0) and after 45 days (D45). Live *A. muciniphila* Muc^T^ administration significantly increased its abundance compared with both CT and HFD groups, confirming successful colonization. Data are presented as boxplots (A, E) or stacked bar charts (D). Statistical significance was assessed by PERMANOVA for beta diversity (B) and paired comparisons using Kruskal-Wallis post hoc tests (E).

None of the alpha diversity metrics differed significantly between groups (Figure 10A,B). Across all indices (Observed ASVs, Shannon, Simpson, and Faith’s PD), values were broadly comparable between CT, HFD, and HFD + live *A. muciniphila* Muc^T^ mice. Although some metrics showed numerically lower median diversity in the HFD and HFD + live *A. muciniphila* Muc^T^ groups, these differences were not statistically significant, indicating that neither HFD feeding nor live *A. muciniphila* Muc^T^ supplementation markedly altered overall microbial richness or evenness within the duration of the study.

Principal coordinate analyzes based on multiple distance metrics confirmed a strong effect of diet on gut microbiota composition. PERMANOVA tests revealed significant separation between CT and HFD groups across all metrics (Bray-Curtis, Jaccard, Unweighted, and Weighted UniFrac; all *p* ≤ 0.05), and similarly between CT and HFD + live *A. muciniphila* Muc^T^ (all *p* ≤ 0.01) (Figure 9B). In contrast, the microbial community composition between HFD and HFD + live *A. muciniphila* Muc^T^ did not differ significantly for any of the tested metrics (*p* > 0.1), although weighted UniFrac showed a slightly higher proportion of explained variance (R² = 0.075), suggesting modest compositional shifts rather than a major restructuring of the community.

Consistent with the diversity analyzes, diet had a major impact on the composition of the gut microbiota, while supplementation with live *A. muciniphila* Muc^T^ induced only limited changes (Figure 10C,D). Across all methods (DESeq2, ALDEx2, and MaAsLin2), multiple taxa differed significantly between CT and HFD groups (17–56 features depending on the method), as well as between CT and HFD + live *A. muciniphila* Muc^T^ (13 - 53 features). In contrast, very few taxa were significantly altered between HFD and HFD + live *A. muciniphila* Muc^T^ (0-2 depending on the method).

Among the taxa most consistently affected by the HFD were members of the *Lachnospiraceae*, *Ruminococcaceae*, and *Erysipelotrichaceae* families, which generally decreased under HFD conditions, while *Desulfovibrionaceae* and *Clostridiaceae* were enriched. Supplementation with live *A. muciniphila* Muc^T^ did not globally restore the HFD-associated microbial profile, but specific taxa responded to the treatment. In particular, ASV242, assigned to *Akkermansia muciniphila*, markedly increased in relative abundance, and also confirmed by qPCR thereby showing successful administration and potential colonization (Figure 10D,E). A single *Lachnospiraceae* amplicon sequence variant (ASV1137, unclassified genus) also showed a modest increase, suggesting a possible secondary response of butyrate-producing commensals to *A. muciniphila* supplementation. Nevertheless, these changes were limited in scope, indicating that live *A. muciniphila* Muc^T^ primarily affects a narrow subset of taxa without broadly reversing the HFD-induced shifts in microbial composition.

Overall, these analyzes show that while the HFD substantially influenced gut microbiota composition compared to controls, supplementation with live *A. muciniphila* Muc^T^ induced only subtle compositional shifts without significant changes in within-sample diversity.

## Discussion

Our study aimed to elucidate the role of live *A. muciniphila* Muc^T^ in modulating metabolic alterations induced by HFD and its influence on intestinal mucus production, glycosylation, and secretion. Our findings confirmed that live *A. muciniphila* Muc^T^ supplementation counteracts obesity-related metabolic disturbances while also significantly impacting changes in mucus composition.

Consistent with previous studies,^8^^,^^9^^,^^15^^,^^22^ our results demonstrate that live *A. muciniphila* Muc^T^ administration mitigates HFD-induced obesity. Mice supplemented with live *A. muciniphila* Muc^T^ exhibited lower body weight gain, reduced fat mass accumulation, and decreased adipose tissue weights across multiple depots (epididymal, subcutaneous, visceral, and brown adipose tissue). Muscle mass remained unaffected, indicating a specific effect on adiposity. These findings support prior evidence from both rodent and human studies showing that *A. muciniphila* supplementation improves metabolic health by enhancing insulin sensitivity and reducing markers of inflammation and liver dysfunction.^8^^,^^9^^,^^15^^,^^22^^,^^23^ In this study we expanded on earlier preclinical results that showed that *A. muciniphila* increased expression of tight junction genes.^8^^,^^9^

By analyzing a large set of antimicrobial peptide production and mucus-related genes in HFD-fed mice it was revealed that live *A. muciniphila* Muc^T^ enhances intestinal function. Notably, the expression of the antimicrobial peptide *Pla2g2a* was significantly upregulated in the jejunum, suggesting a role for live *A. muciniphila* Muc^T^ in stimulating mechanisms of gut defense, potentially supporting gut homeostasis.^24^^,^^25^ However, as this effect was also observed in HFD-fed mice, its specific role in *A. muciniphila-*mediated gut protection remains uncertain. Nevertheless, these observations reinforce prior findings that *A. muciniphila* contributes to gut barrier integrity through antimicrobial peptide regulation and enteroendocrine signaling.^12^

In line with earlier work,^8^^,^^9^^,^^13^ we observed that live *A. muciniphila* Muc^T^ modulates goblet cell differentiation and mucus production. The upregulation of *Math1* in the cecum and jejunum suggests enhanced differentiation of intestinal stem cells into secretory lineages, including goblet cells.^17^ Moreover, live *A. muciniphila* Muc^T^ normalized the HFD-induced dysregulation of the expression of key goblet cell regulators in the jejunum (*Klf4* and *Hes1*), supporting its role in restoring goblet cell homeostasis. These changes likely contribute to improved mucus secretion, consistent with previous studies in rodents showing enhanced mucus layer thickness following *A. muciniphila* supplementation.^8^^,^^9^^,^^13^^,^^26^^,^^27^

Our study provides novel insights into how live *A. muciniphila* Muc^T^ influences mucin production and glycosylation. In the cecum, live *A. muciniphila* Muc^T^ supplementation tended to restore the (non-significant) HFD-induced decrease in *Muc2* and *Muc3* expression. In the colon, we observed a non-significant increase in *Muc13* expression and a significant increase in *Muc1* and *Muc3* expressions.

These transmembrane mucins play critical roles in protecting the gut epithelium and mediating host-microbiota interactions.^28‐30^ Additionally, live *A. muciniphila* Muc^T^ altered glycosyltransferase expression and reversed HFD-induced changes in mucin glycan profiles.^3^^,^^31^

Beyond structural changes, live *A. muciniphila* Muc^T^ also modulated markers involved in mucus secretion and stabilization. Together, these results indicate that live *A. muciniphila* Muc^T^ exerts multifaceted effects on mucus dynamics.

To assess whether these transcriptomic changes were associated with alterations of the mucus composition, we analyzed colonic mucus glycans. Our data revealed that live *A. muciniphila* Muc^T^ supplementation significantly alters glycan abundance and prevalence. Given the role of sialylated and fucosylated glycans in regulating microbial colonization and preventing pathogen adhesion,^3,32‐34^ this modulation may be a key mechanism by which live *A. muciniphila* Muc^T^ enhances gut barrier function and promotes host-microbiota symbiosis. Indeed, sialic acid and fucose sharing by *A. muciniphila* has been proposed to promote abundant butyrate production,^35^ which is known to play a crucial role in maintaining and enhancing intestinal barrier function.^36^ Mucin-type O-glycans act as both structural components of the mucus gel and as information-rich ligands/nutrients that shape mucosal ecology. Terminal sialylation, fucosylation, and sulfation can influence bacterial adhesion (by presenting or masking lectin-binding epitopes), regulate microbial access to underlying glycan cores (via “capping” effects), and modify mucus physicochemical properties (charge, hydration, and resistance to enzymatic degradation), thereby contributing to barrier function.^37‐39^ In this context, the selective modulation of the sialylated structure Neu5Ac2HexNAc3Gal2GalNAc suggests a targeted remodeling of terminal sialic-acid containing epitopes rather than a uniform shift across the glycome (Figure 8). Disialylation may plausibly affect host-microbe interactions through at least two non-mutually exclusive routes: (i) by altering “capping” of mucin chains and thus the pool of free sialic acid available for downstream cross-feeding (with potential consequences for butyrate-producing consortia), and/or (ii) by changing the repertoire of sialylated ligands that engage sialic-acid binding lectins implicated in immune regulation at mucosal surfaces.^35^^,^^40^^,^^41^

Future work will directly test these hypotheses using mucin-binding/adhesion assays, targeted measurement of mucosal sialidase/fucosidase activity, and co-culture systems that quantify whether glycan decapping promotes cross-feeding to butyrate producers.

The ability of live *A. muciniphila* Muc^T^ to change glycan composition supports its role in counteracting diet-induced alterations.^33^^,^^35^ Live *A. muciniphila* Muc^T^ is associated with region-specific shifts in transcripts related to goblet cell biology and mucin handling. However, alcian blue histology did not reveal a change in overall mucus area, suggesting that glycan composition may be altered without a measurable change in bulk mucus thickness. We may not exclude changes in mucus penetrability without a measurable shift in thickness as previously observed in our previous study.^4^

Notably, we may speculate that its unique modulation of specific glycan structures, though low in abundance and unaffected by either control or HFD, highlights a distinct and potentially independent mechanism that may further enhance gut barrier integrity and metabolic health. Future studies should focus on the precise interactions between *A. muciniphila* and host mucins to further elucidate its therapeutic potential in obesity and related metabolic disorders.

**Figure 11.**
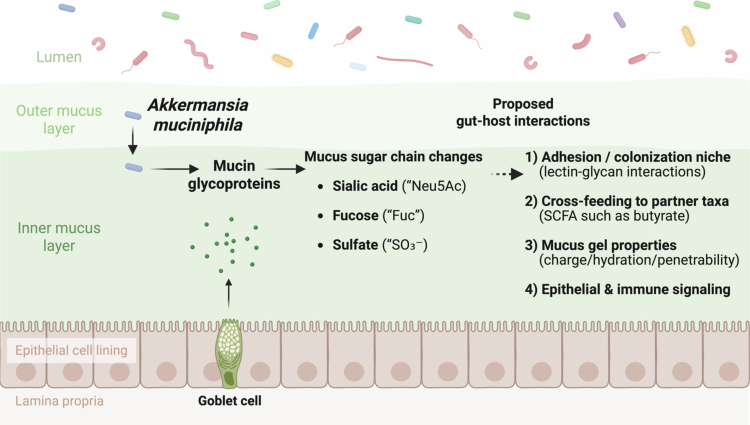
Conceptual model linking mucin sugar-chain remodeling to host–microbe interactions in the colonic mucus niche. Goblet cells secrete mucin glycoproteins that form an inner and outer mucus layer separating the epithelium from the luminal microbiota. In this study, mass spectrometry-based mucin glycomics revealed selective changes in terminal mucus sugar-chain features, including sialylation (Neu5Ac), fucosylation (Fuc), and sulfation (SO₃⁻). These alterations are proposed to influence host-microbe interactions by shaping (1) microbial adhesion and colonization niches through lectin-glycan interactions, (2) nutrient sharing and cross-feeding among mucus-associated taxa with downstream production of metabolites such as short-chain fatty acids (SCFAs; e.g., butyrate), (3) mucus gel properties (charge, hydration, and penetrability), and (4) epithelial and immune signaling. This schematic summarizes testable hypotheses derived from the observed glycan shifts and does not imply direct causality. Created in BioRender. Van Hul, M. (2026) https://BioRender.com/12kaz7q.

Overall, our study advances the current understanding of how live *A. muciniphila* Muc^T^ counteracts HFD-induced metabolic dysregulation. To our knowledge, this is the first study to integrate regional expression of gut markers with glycan composition profiling in the context of live *A. muciniphila* Muc^T^ supplementation. Therefore, we added a schematic model figure illustrating how the observed O-glycan structural shifts could modulate microbial adhesion, mucus barrier properties, and downstream host responses (Figure 11). The ability of live *A. muciniphila* Muc^T^ to restore mucus production, modulate glycan composition suggests that it may serve as a promising therapeutic intervention for obesity and related metabolic disorders.

Consistent with its known sensitivity to dietary modulation, the gut microbiota responded strongly to HFD feeding, whereas supplementation with live *A. muciniphila* Muc^T^ exerted only modest compositional effects.^8^^,^^42^ Although alpha diversity metrics remained unchanged across all groups, principal coordinate analyzes revealed distinct clustering between control and HFD-fed mice, confirming the profound influence of diet on overall microbial structure. In contrast, the microbial communities of HFD and HFD + live *A. muciniphila* Muc^T^ mice largely overlapped, indicating that the beneficial metabolic and mucosal effects observed were not driven by broad restoration of microbial diversity but rather by targeted modulation of specific taxa. Indeed, the marked enrichment of *A. muciniphila* sequences following supplementation demonstrates successful colonization and supports a direct, potentially host-mediated mechanism of action. The modest increase in a single *Lachnospiraceae* ASV further suggests that *A. muciniphila* may foster localized cross-feeding interactions with butyrate-producing commensals, contributing to improved mucosal health without large-scale microbiota restructuring. These findings align with the existing evidence that *A. muciniphila* acts more as a functional keystone species than a dominant ecosystem modulator, exerting metabolically meaningful effects on host physiology.^8^^,^^11^

Nevertheless, some limitations should be acknowledged. Indeed, although we detected region-specific changes in the expression of barrier-associated pathways (e.g., goblet-cell regulators, antimicrobial peptides) together with shifts in mucin glycan composition, our evidence is transcriptional/glycomic in nature (i.e., qPCR and MS). Finally, we note that mechanistic follow-ups (e.g., conditional knockouts, glycosylation inhibitors, or ex vivo organoids) can themselves perturb baseline mucus biology, immune tone, and host-microbe interactions, introducing model-specific confounders.^43^^,^^44^ Plasma LPS concentrations did not differ significantly between groups (Supplementary Figure 1). However, this result should be interpreted cautiously, as circulating basal LPS levels were relatively high in the control mice (i.e., potential contamination during sampling) and were measured as general concentration (ng/ml) and may not fully capture endotoxic activity (EU/ml) changes or intestinal permeability since the concept of metabolic endotoxemia expands beyond LPS load to include LPS characteristics which dictates the effect of metabolic endotoxemia.^45^ Indeed, our recent findings have shown that the lipooligosaccharide (LOS) of *A. muciniphila* exhibits anti-inflammatory properties and can modulate host immune responses.^46^ Therefore, further functional assays will be required to confirm whether *A. muciniphila* Muc^T^ influences mucosal barrier integrity beyond the transcriptional and glycomic levels observed in this study. Accordingly, future work will prioritize triangulation across complementary systems with isogenic/conditional controls, time- and dose-resolved designs (including rescue/reversal where possible), and orthogonal functional read-outs (e.g., FITC-dextran, Ki67, tight-junction staining, mucus penetrability) to establish causality while minimizing bias. In addition, we focused on conventionally colonized mice to avoid the profound baseline alterations in mucus biology induced by antibiotics or germ-free conditions.^20^ Moreover, we used 2 × 10^8^ CFU/day, a dose previously reported as minimally effective for metabolic endpoints in HFD models.^8^^,^^9^^,^^13^ However, whether mucin glycan remodeling follows a graded dose-response, a threshold effect (e.g., requiring sufficient mucosal colonization/enzyme activity), or a time-dependent window remains unknown. Future work should implement dose-gradient designs (e.g., 10^7^-10^9^ CFU/day) combined with time-resolved sampling of mucin glycomics and mucus penetrability to identify the minimum dose and intervention window required to elicit specific glycan shifts. Also, the variability in *A. muciniphila* level of colonization observed in humans^16^ suggests that personalized microbiota-targeted approaches may be necessary to fully harness its therapeutic potential.

While our data support mucus composition and mucin glycosylation as a plausible mechanistic axis, *A. muciniphila* may also influence host metabolism through additional routes (e.g., SCFA-mediated cross-feeding, bile acid signaling, and immune modulation).^12^ Because these pathways were not systematically profiled in the present study, we interpret mucus glycan remodeling as a key focus rather than the sole mechanism. Future studies integrating mucin glycomics with SCFA, bile acid profiling and mucosal immune readouts will be important to quantify their relative contributions.In conclusion, our study highlights the multifaceted role of *A. muciniphila* in modulating mucus structure and alleviating HFD-induced metabolic disturbances. These findings support its development as a promising therapeutic strategy for obesity and related metabolic disorders, warranting further investigation into its host- and microbiota-mediated mechanisms of action.

## Material and methods

### Mice and diets

Seven-week-old male C57BL/6J mice (Janvier, Le Genest-Saint-Isle, France) were housed in pairs under Specific and Opportunistic Pathogen Free (SOPF) conditions, in a controlled environment (22 ± 2 °C, 12‑h light/dark cycle) with ad libitum access to food and water. Upon arrival, the mice were allowed a one‑week acclimatization period, during which they were fed a standard control diet (AIN93Mi, Research Diet, New Brunswick, NJ, USA).

A set of 36 mice was randomly divided into 3 groups of 12 mice: 1) CT group, fed a control diet 2) HFD group, fed a high-fat diet (60% fat and 20% carbohydrates (kcal/100g), D12492, Research diet, New Brunswick, NJ, USA), and 3) HFD + live Akk group, fed a HFD diet and supplemented daily by oral gavage with 2 × 10^8^ CFU/180 μl of live *A. muciniphila* Muc^T^ (ATCC BAA-835) in sterile PBS containing 2.5% glycerol, a dose previously reported as minimally effective in HFD models.^8^^,^^9^^,^^13^ To control for the oral gavage procedure in the test group, the CT and HFD group received a daily dose of vehicle solution (180 µl of PBS containing 2.5% glycerol) by oral gavage. The procedure lasted for 6 weeks.

Body weight was measured three times per week, and body composition was assessed once per week using a 7.5‑MHz time‑domain nuclear magnetic resonance scanner (LF50 minispec, Bruker, Rheinstetten, Germany). All mouse experiments were approved by the local ethics committee (approval number: 2022/UCL/MD/57) and conducted in strict accordance with its guidelines. Housing conditions complied with the Belgian Law of 29 May 2013 on the protection of laboratory animals (agreement number: LA1230314).

### Tissue sampling

At the end of the study (week 6), following a 6‑hour fasting period, all mice were anesthetized with isoflurane (Forene®, Abbott, Queenborough, Kent, England) and then euthanized by cervical dislocation. The epididymal, subcutaneous, visceral, and brown adipose tissue depots, as well as the tibialis anterior and gastrocnemius muscles, and segments of the intestine (jejunum, ileum, cecum, and colon) were collected, weighed, and immediately flash‑frozen in liquid nitrogen before being stored at −80 °C for future analyzes. Additionally, a section of the colon from each mouse was opened (without prior flushing), and the mucus layer was gently scraped with a microscope slide for subsequent glycan composition analysis.

### RNA preparation and gene expression analysis by real-time qPCR analysis

Total RNA extraction and primer sequences as well as method used for the targeted mouse genes are described and published in Paone et al.^4^^,^^5^

### Gene expression heatmap

Gene expression heatmap was created in RStudio version 2023.6.1.524 using the packages readxl (version 1.4.5), ComplexHeatmap (version 2.18.0), circlize (version 0.4.16) and dplyr (1.1.4).^47‐51^

### Mucin glycan extraction and composition

Colonic mucus was suspended in 400 μl mucin extraction buffer (0.2 M Tris, pH 8, 1% SDS, 10 mM DTT). The samples were incubated at 60 °C for 90 min. Iodoacetamide was added from a 1 M stock solution to a final concentration of 100 mM. The samples were incubated at RT for 90 min in the dark. The reduced samples were spin filtered through a 100k MWCO amicon 0.5 filter (merck) for 15 min at 14000 g. Lithium dodecyl sulfate (LDS) loading buffer (10 μl; Thermo Fisher) was added to the samples and loaded onto a 1% vertical agarose gel cast in Tris-Glycine-SDS (TGS) buffer (Biorad). Vertical agarose gel electrophoresis (VAGE) was carried out at 100 V for 45 min. The mucins/proteins were transferred onto Immobilon Psq (Merck) in Tris-glycine^52^ buffer, using Trans-blot Turbo (25 V, 1 A, 60 min; Biorad). The region of the blot were mucins migrated was cut out and the blot was immersed into 500 μl 0.5 M NaBH4 in 0.05 M NaOH. The *β*-elimination reactions were incubated at 45 °C for 16 h and quenched by the stepwise addition of 1 ml 5% aqueous acetic acid. The samples were desalted on in-house prepared cation exchange columns using Amberlite 50Wx8 H + 200-400mesh. The samples were dried under vacuum and removal of borates was carried out with co-evaporation with methanol under nitrogen.

For the base required for permethylation, 400 μl of 50% NaOH were mixed with 800 μl dry MeOH and 4 ml of anhydrous DMSO. The resulting gel was washed 5 times with 4 ml DMSO before resuspending in 4 ml DMSO. The dried samples were dissolved in 100 μl anhydrous DMSO, followed by the addition of 150 μl of the prepared base and 75 μl of iodomethane. The samples were vortexed for 2 h at 2000 rpm and the reactions were quenched by the addition of 500 μl H2O. Excess of iodomethane was removed with a flow of nitrogen.

The permethylated glycans were loaded onto a Swift-HLB cartridge (Merck). Salts and other hydrophilic contaminants were removed with 4 × 1 ml washes with H2O and permethylated glycans were eluted with 4 × 1 ml of MeOH. The eluted glycans were dried under vacuum and redissolved in 10 μl of 30% acetonitrile in 0.1% aqueous trifluoroacetic acid (TA30). The sample (0.5 μl) was mixed with 0.5 μl of 2,5-dehydroxy-benzoic acid (DHB, 20 mg/ml in TA30) and spotted onto a MTP ground steel MALDI target plate. The samples were analyzed by MALDI-ToF MS on a Bruker Autoflex in positive reflectron mode. Peak detection and integration in the mass spectra was done using flexAnalysis (v3.4, Bruker Daltonics) with the following settings: Peak detection algorithm was Snap2, signal to noise threshold = 2, relative intensity threshold = 0, minimum intensity threshold = 2, SNAP2 average composition was set to “sugar”, baseline subtraction was set to TopHat. Relative abundance of each peak identified as glycan was calculated as the area of the peak over the sum of all peaks that were identified as glycans. Only glycans present in at least 3 mice and in at least one group were shown.

### Lipopolysaccharide assay

Mouse portal vein serums were prepared as previously described.^53^ To disperse endotoxin molecules, serums were diluted (1:10) in biodispersing agent (PYROSPERSE, Lonza, Bales, Switzerland). The samples were then heated at 70 °C for 15 min to inactivate nonspecific endotoxin inhibitors. LPS quantification with a competitive inhibition enzyme immunoassay (Cloud-Clone Corp, Houston, TX) was done using the manufacturer instructions. Optical density was measured on a spectrophotometer at 450 nm and LPS concentration was calculated with the standard curve.

### Histology

Colon segments were prepared and stained with alcian blue as previously described.^4^^,^^5^ Images were captured on a 3DHistech Panoramic SCAN II and analyzes were performed in a blinded manner on 2 non-consecutive slices. Representative images were obtained at a 20x magnification on the same software.

Mucus thickness analyzes were performed using 3DHistech slide viewer 2.9.0 software. Two experimenters annotated a minimum of 180 different measurements per slides for a total of 15 696 measurements.

Goblet cells quantification was performed using QuPathv0.6.0 the luminal side, muscularis mucosae, submucosa and muscle layer were removed and the total area was measured.^54^ The software was trained to quantify the blue (alcian blue; mucus) on 2 training slides and the same parameters were applied to the images dataset. The proportion of goblet cells is inferred based on the ratio of blue staining over the total area.

### Bacterial DNA extraction

Genomic DNA was extracted on fecal samples collected at the beginning (day 0) and at the end of the study (day 45) and kept at −80 °C until use. Extraction was performed using a QIAmp DNA Stool Mini Kit (Qiagen, Hilden, Germany), according to the manufacturer’s instructions after a bead-beating step.

### Quantitative PCR for *Akkermansia muciniphila*

Relative abundance of *Akkermansia muciniphila* was carried out by qPCR with bacterial primers (F: 5'- CAGCACGTGAAGGTGGGGAC -3', R: 5'- CCTTGCGGTTGGCTTCAGAT -3'), with the Quantstudio3 system and software (Applied Biosystems, Den Ijssel, The Netherlands) using the GoTaq qPCR sybr green mix (Promega, Madison, Wisconsin, USA), according to the manufacturer’s instructions. All samples were run in duplicate in a single 96-well reaction plate. The cycle threshold of each sample was compared with a standard curve made by serially diluting genomic DNA isolated from a pure culture of the type strain *Akkermansia muciniphila* (DSM 06-14) (BDSMZ, Braunschweig, Germany).

### 16S rRNA gene sequencing and preprocessing

Demultiplexed paired-end FASTQ files were analyzed using QIIME2 (amplicon-2024.10) on an Apple M2 Max system. Sequencing was performed by MrDNA (Shallowater, TX, USA) using modified V4 primers (515F: GTGYCAGCMGCCGCGGTAA; 806 R: GGACTACNVGGGTWTCTAAT). Reads were imported into QIIME2 and summarized to verify sequence integrity and quality. Primer sequences were removed, and read quality was inspected through interactive quality plots.

### Denoising, filtering, and taxonomic assignment

Paired-end reads were denoised and merged using the DADA2 algorithm implemented in QIIME2 to generate amplicon sequence variants (ASVs).^55^^,^^56^ Truncation and trimming lengths were optimized based on per-base quality scores to maximize sequence retention. Low-abundance ASVs (<5 total counts across all samples) were excluded. Chimeric sequences were identified and removed during DADA2 denoising.^57^ Taxonomic classification was performed using a pre-trained SILVA 138 classifier, trimmed to the 515F/806R region. Non-bacterial sequences (chloroplasts, mitochondria, and eukaryotes) were filtered out prior to downstream analysis.

### Phylogenetic reconstruction and diversity analysis

Aligned representative sequences were generated using MAFFT, and a mid-point rooted phylogenetic tree was built with FastTree2. Alpha and beta diversity analyzes were conducted in QIIME2 and R using exported feature tables and phylogenetic trees. Alpha diversity metrics (Observed ASVs, Shannon, Simpson, and Faith’s PD) were calculated from rarefied data (rarefaction depth = 15,000 reads). Beta diversity was assessed using Bray–Curtis, Jaccard, weighted and unweighted UniFrac distance metrics. Differences in community composition between groups were evaluated by PERMANOVA (999 permutations) using the adonis2 function in the vegan R package.

### Differential abundance analysis

Taxonomic and abundance tables were imported into R (version 4.4.1) and analyzed through a modular Quarto pipeline. Differential abundance was assessed using DESeq2, ALDEx2, and MaAsLin2 to identify taxa consistently associated with experimental groups. Multiple testing correction was performed using the Benjamini–Hochberg FDR procedure (q < 0.05). Results across methods were integrated to identify reproducibly differentially abundant taxa. Heatmaps, bar plots, and volcano plots were generated using ggplot2 and ComplexHeatmap packages.

### Statistical and bioinformatics analysis

Statistical analyzes were conducted using GraphPad Prism version 10.4.2 for macOS (GraphPad Software, San Diego, CA, USA). A mixed-effects model (REML) was employed to assess the effects of treatment and gut segment or time across repeated measures, using a stacked matching structure and without assuming sphericity. Fixed effects were analyzed using type III tests, and Geisser-Greenhouse correction was applied when necessary. The alpha level was set at 0.05. Multiple comparisons within each gut segment were performed using Tukey’s post hoc test. Data are presented as mean ± standard error of the mean (s.e.m.). Quantitative PCR (qPCR) analysis was performed to assess gene expression across different gut segments, using RPL19 as the reference housekeeping gene for normalization. A mixed-effects model (REML) was applied with stacked matching to account for repeated measures within individual animals and to evaluate the fixed effects of gut segment, treatment (CT, HFD, HFD + live Akkermansia), and their interaction. The assumption of sphericity was not applied, and Geisser-Greenhouse’s epsilon correction was used. Post hoc Tukey’s multiple comparisons were conducted within each gut segment to compare treatments. To ensure data integrity, the ROUT (Robust regression and Outlier removal) test was applied to identify and exclude statistical outliers. As a result, the number of mice analyzed may vary between groups and parameters. The results were considered statistically significant at *P* < 0.05.

## Supplementary Material

Supplementary Figure 1.pdf Supplementary Figure 1.pdf

## Data Availability

All R-based analyzes were performed within a reproducible Quarto document (MNUT_Pipeline_220925L.qmd) combining code, output, and narrative. Figures were created in R and GraphPad Prism v10. The raw amplicon sequencing data analyzed in this study have been deposited in the European Nucleotide Archive (ENA) at EMBL-EBI under accession number PRJEB102010 (https://www.ebi.ac.uk/ena/browser/view/PRJEB102010). The mass spectrometry glycomics data have been deposited via GlycoPOST^58^ under the number ID: GPST000637 (https://glycopost.glycosmos.org/GPST000637).
