# Supplementary material for: Akkermansia muciniphila modulates intestinal mucus composition to counteract high-fat diet-induced obesity in mice
Source: Gut Microbes. 2026 Jan 9;18(1):2612580. doi: 10.1080/19490976.2025.2612580 (PMC12795273; doi:10.1080/19490976.2025.2612580)
Supplement: Supplementary Figure 1.pdf [file KGMI_A_2612580_SM9910.pdf]

A

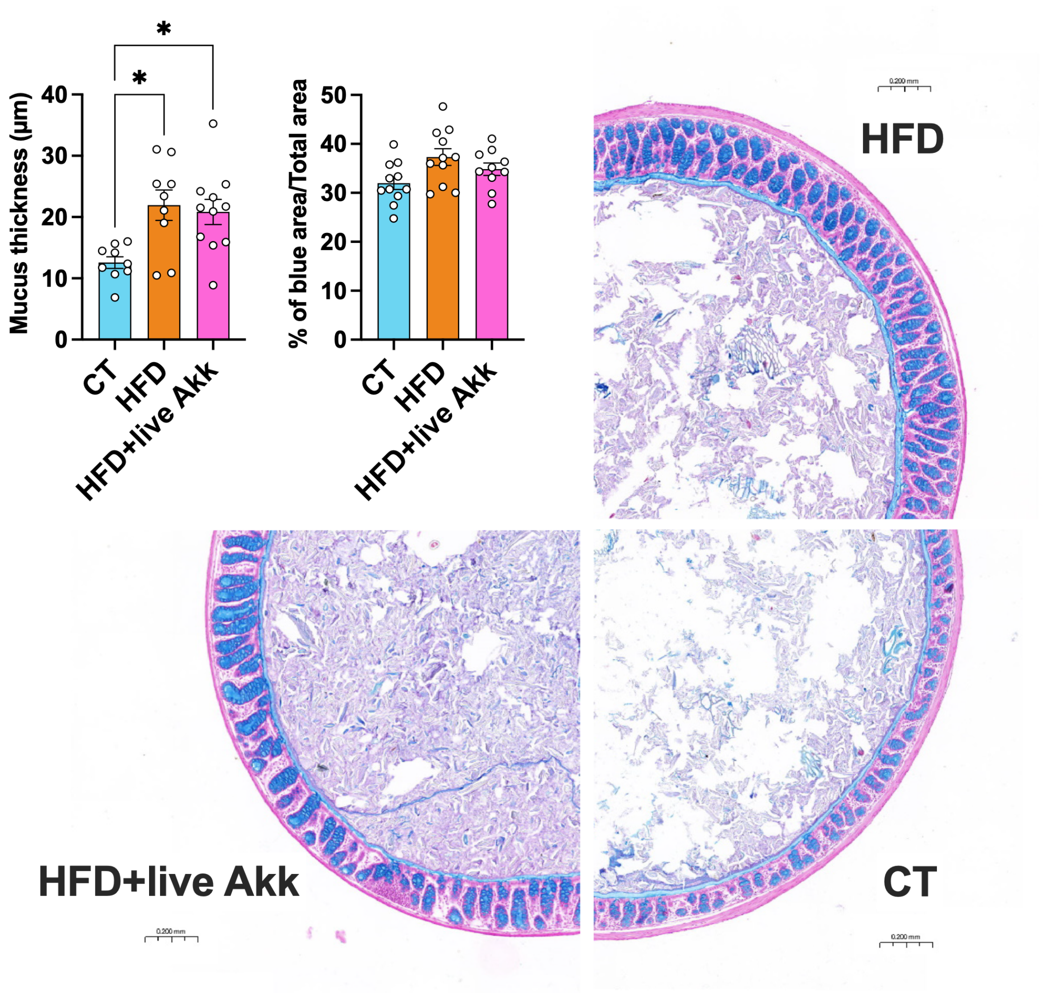

B

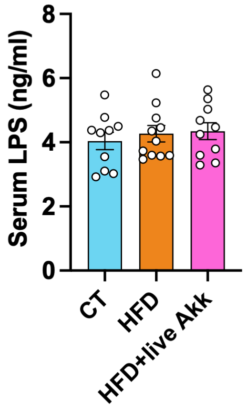

Supplementary Figure 1. Mucus thickness and serum LPS

(A) Representative images for each group and mucus thickness measured in micrometers in the proximal colon, percentage of blue area on the total mucosal area in the proximal colon (n=9-11/group). (B) Portal vein serum LPS concentration was quantified by a competitive inhibition enzyme immunoassay. Data are means ± s.e.m (n=10-11/group. Kruskal-Wallis followed by Dunn's test were applied based on data distribution. The presence of outliers was assessed using the Rout test. *P < 0.05.
